# Central precocious puberty in Boston boys: A 10-year single center experience

**DOI:** 10.1371/journal.pone.0199019

**Published:** 2018-06-27

**Authors:** Lisa Swartz Topor, Kimberly Bowerman, Jason T. Machan, Courtney L. Gilbert, Tairmae Kangarloo, Natalie D. Shaw

**Affiliations:** 1 Department of Pediatrics, Division of Pediatric Endocrinology, Alpert Medical School of Brown University, Providence, Rhode Island, United States of America; 2 Hasbro Children’s Hospital, Providence, Rhode Island, United States of America; 3 Alpert Medical School of Brown University, Providence, Rhode Island, United States of America; 4 Lifespan Biostatistics Core, Rhode Island Hospital, Providence, Rhode Island, United States of America; 5 Department of Psychology, University of Rhode Island, South Kingston, Rhode Island, United States of America; 6 University of Massachusetts Medical School, Worcester, Massachusetts, United States of America; 7 Reproductive Endocrine Unit, Massachusetts General Hospital, Boston, Massachusetts, United States of America; 8 Clinical Research Branch, National Institute of Environmental Health Sciences, National Institutes of Health, Research Triangle Park, Durham, North Carolina, United States of America; University of Sydney, AUSTRALIA

## Abstract

**Objective:**

Recent studies in the US and abroad suggest that boys are undergoing puberty at a younger age. It is unknown if this secular trend extends to boys with central precocious puberty (CPP), who sit at the extreme end of the pubertal spectrum, and if neuroimaging should remain a standard diagnostic tool.

**Study design:**

Retrospective chart review of all boys with CPP seen by Endocrinology at a US pediatric hospital from 2001–2010.

**Results:**

Fifty boys had pubertal onset at an average age of 7.31 years (95CI 6.83–7.89), though many did not present until nearly one year thereafter, by which time 30% were mid-to-late pubertal. Boys were predominantly non-Hispanic White and 64% were overweight/obese. The majority (64%) of boys had neurogenic CPP (CNS-CPP) with neurofibromatosis type I being the most common diagnosis. Diagnosis of CPP led to discovery of a neurogenic lesion in only 3 of 32 (9%) CNS-CPP cases. The remaining boys, with idiopathic CPP (36%), were indistinguishable from those with CNS-CPP aside from four boys who endorsed a family history of PP (22% vs. 0% among CNS-CPP cases). Importantly, there was no change in the incidence of male CPP after accounting for the increase in clinic volume during this time period.

**Conclusion:**

In this contemporary Boston-based cohort of 50 boys with CPP, most cases were neurogenic, consistent with older literature. Several idiopathic cases had a family history of PP but were otherwise indistinguishable from CNS-CPP cases. Thus, neuroimaging remains a critical diagnostic tool. We find no evidence for an increase in the prevalence of male CPP.

## Introduction

The first sign of male puberty is an increase in testicular volume (>3 ml) which signals the re-activation of the neuroendocrine components of the reproductive axis. While pubertal onset in boys has historically occurred at an average age of 11.6 ± 0.09 years [1], recent large-scale observational studies in the US [2], Europe [3], and Asia [4] suggest that puberty may now be occurring earlier in boys, akin to the pattern observed in contemporary girls. In girls, the obesity epidemic and endocrine disrupting chemicals are thought to be the main drivers of early puberty (reviewed in [5, 6]), yet perplexingly, these same environmental factors have often been associated with delayed rather than earlier puberty in boys [7–11], suggesting there may be different biological mechanisms underlying changes in the timing and tempo of male puberty.

The diagnosis of precocious puberty (PP) in males, as in females, is based entirely on a statistical definition equal to pubertal onset at an age that is two or more standard deviations below the mean, or puberty before age 9 years in boys. The majority of cases of PP reflect premature activation of the neural components of the male reproductive axis (central PP, CPP), which leads to an increase in gonadotropins, testicular volume, and testosterone production, as opposed to a peripheral or exogenous stimulus or source of androgens (peripheral PP). CPP in a boy is frequently caused by a pathological brain lesion, whereas CPP in girls is most often idiopathic. Clinical guidelines for the evaluation of male CPP, which include neuroimaging to rule-out intracranial malignancy, are strongly tied to this age-based, statistical criterion.

There has been very little investigation into whether there has been a rise in the incidence of male CPP over the past decade [4, 12–14] and no studies have been conducted in US boys. If there has been a population-level shift in the timing of male puberty, one would expect more boys to meet CPP criteria, more referrals for endocrine evaluation, and potentially more (but unnecessary) testing and neuroimaging. To begin to fill this knowledge gap, we determined the incidence and underlying etiology of male CPP over a 10-year period at a leading US pediatric tertiary care center.

## Materials and methods

We conducted a retrospective chart review of all male patients seen in the Pediatric Endocrinology Division at Boston Children’s Hospital (BCH) between 2001–2010 with an International Statistical Classification of Diseases 9^th^ edition (ICD-9) code for PP (ICD-9 259.1). Note that because different billable medical codes were typically used for related diagnoses including familial male-limited PP (e.g. billed as testicular hyperfunction), androgen-secreting tumors (e.g., billed as neuroendocrine tumor), and congenital adrenal hyperplasia (e.g., billed as adrenogenital disorder), such patients are absent from the current study by design. Complete medical records were reviewed by trained study staff using a standardized abstraction form to confirm the diagnosis of CPP or to assign an alternative diagnosis (e.g. premature adrenarche, early-normal puberty, pubic hair of infancy). CPP was defined as a testicular volume > 3 mL, *and/or* a baseline luteinizing hormone (LH) ≥ 0.3 IU/L, *and/or* LH > 5 IU/L after leuprolide stimulation (10 μg/kg administered subcutaneously) in a boy younger than 9 years old [15]. CPP cases were sub-categorized as either neurogenic (CNS-CPP), which encompassed cases related to a brain injury, brain tumor, hydrocephalus, or a known syndrome, or as idiopathic CPP (iCPP). Medical, family, and social history, physical exam findings, and laboratory and imaging results were recorded. Bone age was assigned by a pediatric endocrinologist using the method of Greulich and Pyle [16]. A patient was said to have a family history of early puberty if his mother reported menarche before age 10 years or if his father reported an early growth spurt relative to peers. All data collection forms were reviewed by at least one pediatric endocrinologist (L.S.T and N.D.S). Both endocrinologists reviewed a subset of forms (n = 10) and demonstrated 100% inter-rater reliability in the selected diagnosis.

To discern whether any observed change in the incidence of male CPP between 2001 and 2010 might represent a true change in disease incidence or reflect an increase in total referrals to BCH, we also used the Informatics for Integrating Biology and the Bedside (i2b2) [17] computational tool to query the BCH electronic medical record and determine the number of male patients seen per year in the BCH Endocrinology clinics.

Reproductive hormone assays were performed in the BCH Department of Laboratory Medicine. Serum LH and FSH were measured using a chemiluminescence immunoassay manufactured by Bayer Diagnostics (now Siemens, Tarrytown, NY) from 2001–2003 and by Roche Diagnostics (Indianapolis, IN) thereafter. These two assays are highly correlated with minimal bias and differ only in their limit of detection [LOD] (Bayer 0.2 IU/L, Roche 0.1 IU/L). The limits of quantification (LOQ) for LH and FSH are the same as the LOD for these assays. For LH, the inter-assay coefficient of variation (CV) is 3.2% for quality control sera (QCS) containing 1.8 IU/L, and for FSH, the CV is 2.1% for QCS containing 6.8 IU/L. Total testosterone (TT) was measured using one of two in-house assays which were cross-validated: high-performance liquid chromatographic (HPLC) steroid separation followed by immunoassay (2001–2006; LOD 12.5 ng/dL) or an LC-mass spectroscopy assay (2006–2010; LOD 10 ng/dL) which currently has an inter-assay CV of 8.2% for QCS containing 53 pg/mL. Testosterone assays were interconverted according to the formula y = 0.665x + 5.19. Dehydroepiandrosterone sulfate (DHEAS) was measured with an electrochemiluminescent immunoassay. Genetic testing for CPP was not routinely performed in the clinic during this time period.

This protocol was approved by the BCH Institutional Review Board. The requirement for informed consent was waived due to the retrospective nature of the study.

### Statistical analyses

All analyses were conducted using SAS Software 9.4 (SAS Inc., Cary, NC). Baseline characteristics were summarized with means with 95% confidence intervals for continuous variables and frequencies and percentages for categorical variables.

Demographics, history, laboratory studies, and physical exam findings in boys with iCPP and CNS-CPP were compared using generalized linear models. Tanner stages were modeled as binomial proportions. Hormone levels were compared using a generalized Tobit model, which is suitable for censored data, when values fell below the assay LOD. Classical sandwich estimation was used to adjust for any model misspecification. A generalized linear model for binomial data was used to test for a temporal change in the number of male CPP or premature pubarche cases before and after adjusting for the number of male clinic patients. Statistical significance was established at the 0.05 level and all interval estimates were calculated for 95% confidence.

## Results

**Fig 1:**
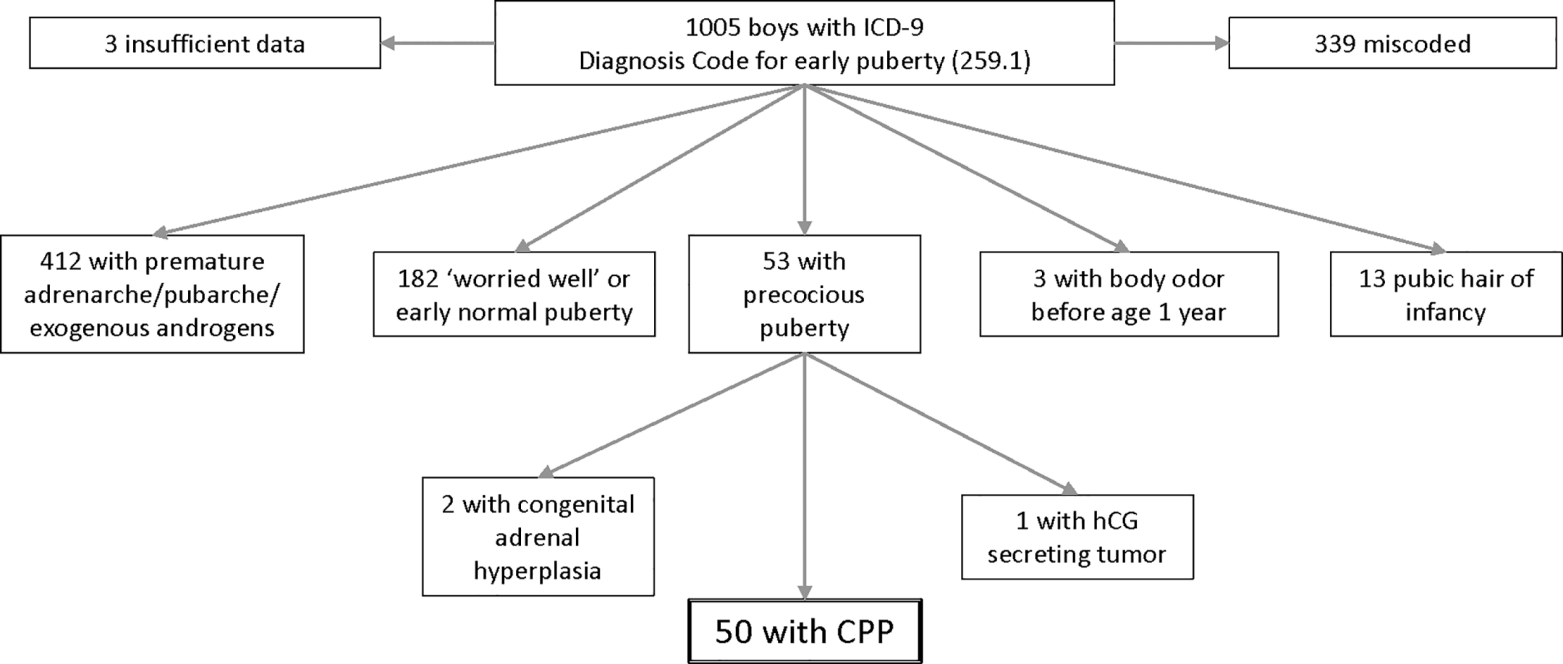
Inclusion flow diagram of the male CPP cohort.

From 2001–2010, 1005 boys were evaluated in the BCH Department of Pediatric Endocrinology and assigned a diagnostic code for PP. Careful review of the medical records revealed that only 53 met formal criteria for PP, whereas the majority of patients with a pubertal diagnosis had either premature pubarche or “early normal” puberty and a small minority were infants with isolated body odor or pubic hair (**Fig 1**). All patients with PP had CPP with the exception of three patients who were excluded from further analyses: one patient with a hypothalamic human chorionic gonadotropin-secreting germ cell tumor, and two patients with CPP triggered by peripheral PP (congenital adrenal hyperplasia), resulting in a final CPP cohort of 50 boys.

The boys with CPP were predominantly non-Hispanic White [NHW] (66% NHW, 10% Hispanic, 8% African-American, 4% Asian, 12% Mixed/Other/Unknown), representative of the demographics of the BCH catchment area. None of the boys were international adoptees. and Patients came to clinical attention at an average age of 8.13 years (95CI 7.65–8.60) (**Table 1**). By parental report, pubertal signs first appeared at an average age of 7.31 years (95CI 6.83–7.79), however, it is quite possible that puberty occurred even earlier as parents frequently underestimate the onset of puberty in their sons [18]. Thirty percent of patients (n = 15) were already mid-to-late pubertal (defined as testicular volume ≥ 8 mL) at presentation. The majority (64%) were overweight or obese at the time of initial endocrine evaluation, and this figure dropped to 48% when bone age, rather than chronologic age, was used to determine BMI percentile [19].

**Table 1:** Baseline characteristics of the cohort.

Clinical Measure	iCPP		CNS-CPP		p-value
	n	Mean (95% CI)	n	Mean (95% CI)	
Age at Pubertal Symptom Onset, Years	18	7.56 (7.07, 8.04)	31	7.17 (6.47, 7.86)	0.36
Age at CPP Diagnosis, Years	18	8.73 (8.30, 9.15)	32	7.79 (7.11, 8.46)	0.02
Height Z-Score	18	1.2 (0.51, 1.90)	29	0.65 (0.07, 1.23)	0.22
Weight Z-score	18	1.65 (1.06, 2.24)	29	1.05 (0.49, 1.61)	0.14
BMI Z-score	18	1.54 (1.19, 1.88)	27	1.25 (0.85, 1.66)	0.29
Pubic hair, Tanner stage	17	T1 11.76%, T2 35.39%, T3 47.06%, T4 5.88%, T5 0%	30	T1 43.33%, T2 26.67%, T3 23.33%, T4 3.33%, T5 3.33%	0.075
Pubic hair present, %	17	88.24 (62.22, 97.16)	30	56.7 (38.37, 73.31)	0.043
Axillary hair present, %	17	41.18 (21.0, 65.0)	25	4.0 (0.5, 25.0)	0.017
Body odor present, %	17	76.5 (50.5, 91.1)	23	43.5 (24.7, 64.3)	0.049
Bone Age—Chronological Age, Years	17	2.61 (1.71, 3.51)	29	1.78 (1.14, 2.40)	0.13
Family History Precocious Puberty, %^#^	17	23.5 (8.8, 49.6)	18	0	—

^#^ Female relative with menarche at or before age 9 years or early puberty reported in a male relative

Nearly 2/3 of boys with CPP had CNS-CPP secondary to a tumor, neuroanatomic abnormality, or genetic syndrome (**Table 2**). The remaining 18 boys, classified as iCPP, were without syndromic traits and either had no intracranial lesion on brain imaging (n = 15) or prolonged follow-up without neurological symptoms (n = 2, 4 and 10 years follow-up), and one boy declined brain MRI after CPP diagnosis and was lost to endocrine follow-up. CNS-CPP and iCPP cases did not differ in anthropometrics, including the percentage of overweight/obesity (43% vs. 56% using bone age, p = 0.5).

**Table 2:** Underlying etiology of CNS-CPP in 32 boys.

Causes of CNS-CPP (n = 32)	n
Neurofibromatosis type 1	10
Optic glioma	4
Hypothalamic hamartoma	3
Other CNS tumor	3
Genetic syndrome*	4
Brain injury	8

*Genetic disorders: Costello Syndrome, Encephalocraniocutaneous lipomatosis, Mucopolysaccharidosis type III [Sanfilippo Syndrome], Wolf-Hirschhorn Syndrome

Neurofibromatosis type 1 (NF-1) in association with an optic or hypothalamic glioma was the most common cause of CNS-CPP. This may reflect the large referral base of the BCH Multidisciplinary Neurofibromatosis Program as similar studies conducted at major university hospitals have also reported a high prevalence of NF-1 and optic gliomas among boys with CNS-CPP [20–22]. The remaining CNS-CPP patients had other brain tumors, brain injury (e.g. congenital hydrocephalus, hypoxic ischemic injury), or a genetic syndrome associated with CPP. In 3 boys (aged 9 months, 7.5 years, and 8.3 years), CPP occurred in an otherwise healthy, asymptomatic patient, thereby prompting neuro-imaging and unmasking neuropathology (**S1 Fig**). These boys were diagnosed with either a hypothalamic or optic pathway tumor and had no distinguishing clinical or laboratory findings.

**Fig 2:**
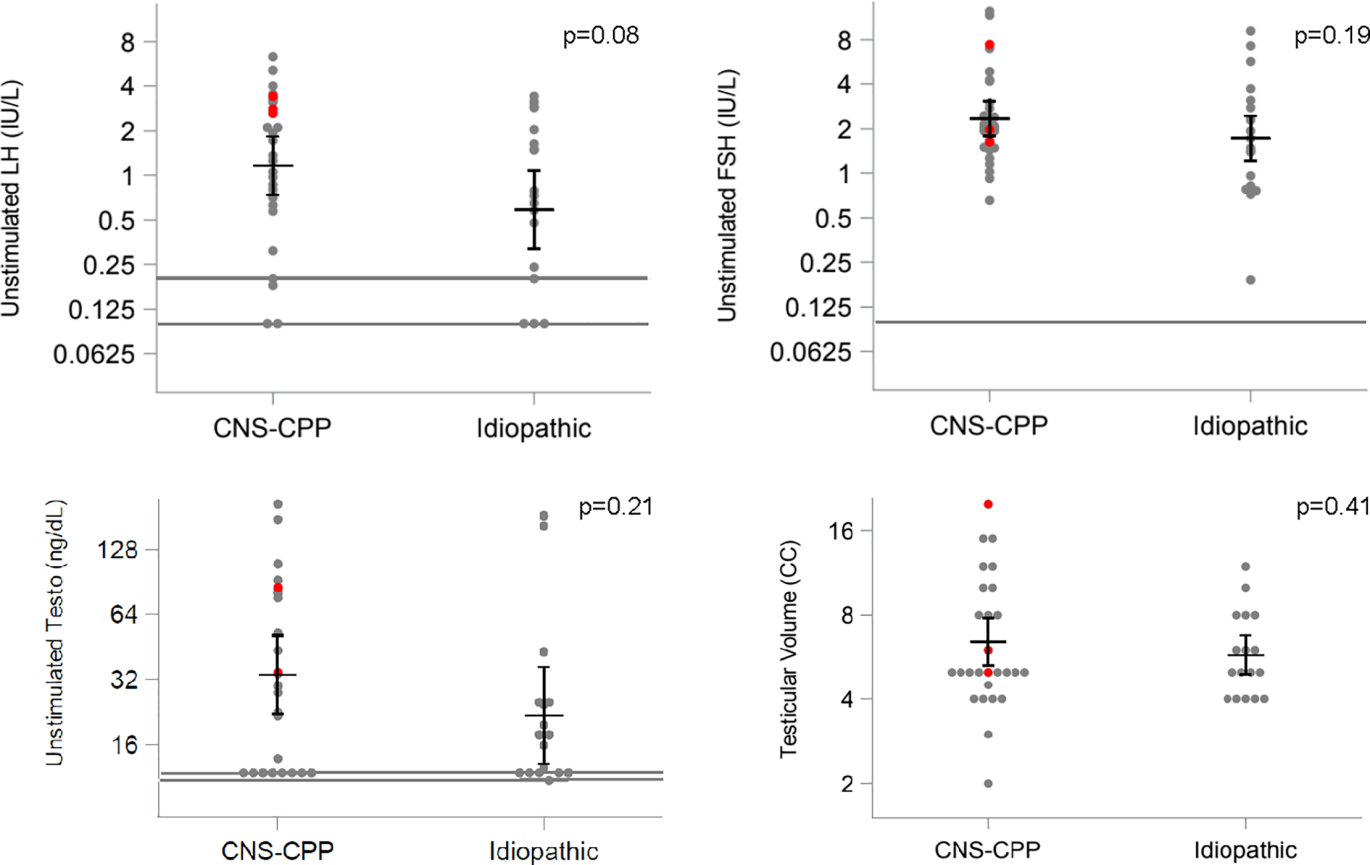
Baseline LH, FSH, and total testosterone (Testo) levels and testicular volume in CNS-CPP and iCPP cases. Means and 95CI overlay raw data points. Red dots represent measures from the 3 patients in whom the diagnosis of CPP unmasked a brain neoplasm. Gray lines represent assay lower limits of detection. P-values reflect group comparisons using generalized linear models. To convert to SI units, multiple Testo by 0.0347.

There were several notable overall differences between boys with CNS-CPP and iCPP. While boys in both groups demonstrated the first signs of puberty at the same age, CNS-CPP patients were diagnosed at a younger age (**Table 1**). Of note, 50% of CNS-CPP boys and 50% of iCPP boys had pubertal onset in the “borderline” age range (8–9 years). Boys with iCPP were more likely to have adult type body odor, axillary hair, and pubic hair, yet there were no group differences in DHEAS levels (iCPP 3.1 μmol/L, 95CI 1.9–5.3 vs. CNS-CPP 1.9 μmol/L, 95CI 1.2–3.0, p = 0.16). CNS-CPP patients, despite being younger, showed a tendency toward higher baseline LH levels (p = 0.08), higher TT levels (p = 0.21), and larger testicular volumes (p = 0.41) (**Fig 2)**. A family history of early puberty was present in nearly one-quarter of iCPP cases but was absent in all CNS-CPP boys. While several paternally imprinted genes have recently been implicated in hereditary and sporadic forms of CPP (e.g. *DLK1*, *MKRN3*) [23–25] in 3 out of the 4 familial CPP cases in the current cohort, it was the mother who reported a history of early menarche, suggesting maternal transmission.

**Fig 3:**
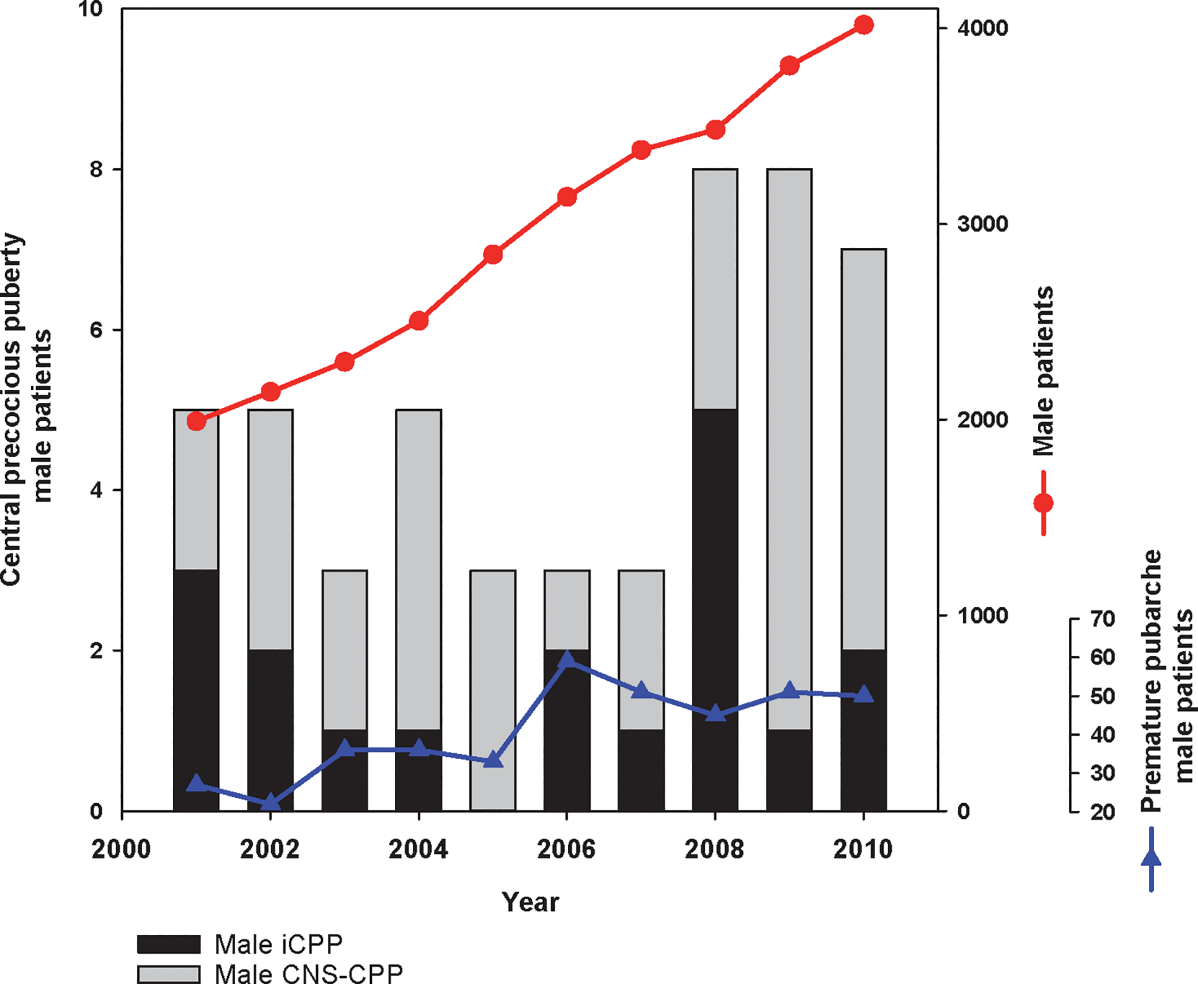
Number of male patients with CPP, male patients with premature pubarche, and male patients (all diagnoses) seen per annum by BCH Endocrinology. Note different y-axes scales.

There was a slight increase in the number of new CPP cases per year from 2001–2010 (7% per year, 95CI 0–13%, p = 0.03). However, over this same timeframe there was a comparable increase in the number of new cases of premature pubarche (8% per year, 95 CI 5–12%, p<0.001; CPP vs. premature pubarche, p = 0.6), a condition not believed to have a neuroendocrine trigger, suggesting there may be an unmeasured confounder. Indeed, there was no change in the number of CPP cases after accounting for the concurrent increase in the number of male patients seen in the Pediatric Endocrine clinic from 2001–2010 (**Fig 3**).

## Discussion

The current study offers a unique window into the male CPP patient population at a major US academic medical center from 2001–2010. Consistent with traditional teaching, the majority of CPP cases in boys were due to neoplastic or neuroanatomic lesions, and boys with CNS-CPP were diagnosed at a younger age than those with an idiopathic cause [12, 26]. An earlier diagnosis in CNS-CPP boys may have been related to closer medical surveillance, as nearly 80% had a known CNS lesion, leading many of the boys to be referred to the Endocrine Clinic for monitoring of hypothalamic-pituitary function prior to the time of CPP diagnosis. Conversely, iCPP boys with a family history of PP may have come to clinical attention later because of parental misconceptions about normal pubertal timing. Importantly, we observed only a minor increase in the number of CPP diagnoses per year which closely mirrored the increase in both premature pubarche and total endocrine referrals. This suggests there was no change in the true incidence of male CPP but rather an increase in overall patient volume.

These results are largely consistent with several recent studies on secular trends in male CPP. A Turkish university hospital study (n = 101 cases) found that CNS-CPP patients were younger at diagnosis than idiopathic cases but were more reproductively mature, as indicated by higher basal TT levels and stimulated LH levels [12]. They reported a slight increase in male idiopathic (but not organic) CPP cases from 2003 to 2009, which was followed by a 5-year plateau, but did not control for changes in overall clinic volume, a critical potential confounder. A Spanish epidemiologic study of CPP reported a small increase in male CPP cases from 1997 to 2009 which coincided with a large demographic shift related to increased immigration and international adoption, two well-recognized risk factors for CPP [27]. A population-level study in South Korea using national registry data also demonstrated a small increase in the incidence of male CPP cases during this timeframe, but the increase was limited to boys ages 8 to 9 years [4]. Lastly, an earlier (1993–2001) Danish registry-based study reported no change in the incidence of PP in boys [28]. Taken together with the current findings, these studies do not provide strong evidence for a change in the incidence of male CPP since the early 2000s.

We had predicted that a population-level shift in the timing of male puberty would lead to more diagnoses of male CPP in the borderline (8-9-year) age range. While 50% of iCPP boys displayed signs of puberty between ages 8 and 9 years, the same was true for boys with CPP triggered by true neuropathology, suggesting that this figure does not reflect a secular change. We also found no difference in the prevalence of overweight/obesity among iCPP and CNS-CPP boys suggesting that obesity was not a primary driver of PP in iCPP boys in this cohort.

While this study has several limitations, including those inherent to a retrospective chart review, we were careful to follow best practice guidelines for medical record review, as previously described [29], to minimize errors and potential biases. This study included a relatively small and homogeneous patient population (non-Hispanic White boys) at a major academic medical center which may limit generalizability to other large university hospital settings. We also used overall clinic volume as a proxy for the hospital catchment population, recognizably the preferred denominator in calculations of incidence rate. In contrast to previous epidemiologic studies where concern has been raised about misclassification bias in male genital Tanner staging [30], we have confidence in the diagnoses of male PP in the current study, as in every case the diagnosis was made by a pediatric endocrinologist skilled in Tanner staging and in the precise measurement of testicular volume using orchidometry. We had limited access to height and weight measurements pre-CPP diagnosis precluding an assessment of the temporal relationship between overweight/obesity and CPP in these boys. Furthermore, as overweight/obesity was defined by BMI, it remains possible that the higher BMIs observed among boys in the current study were inflated by the increase in fat-free (muscle) mass that is known to occur during male pubertal development [31] and do not represent true obesity.

The current study demonstrates the power of a detailed family history in informing the diagnostic workup of male CPP patients. While iCPP and CNS-CPP were indistinguishable on all standard clinical and laboratory measures, the presence of a family history of CPP was a strong predictor of an idiopathic cause. There is currently insufficient evidence to support a change in current guidelines which recommend neuroimaging of all male CPP patients (< 9 years old). As we gain further insight into the genetic architecture of male CPP, which currently includes genes in pathways implicated in delayed puberty and/or congenital GnRH deficiency (e.g., *KISS1*, *KISS1R*, *PROKR2*) as well as novel genes (e.g., *MKRN3*, *DLK1*) [23, 24, 32–35], genetic testing may supersede neuroimaging in standard diagnostic algorithms.

In conclusion, comprehensive medical record review of 50 male CPP patients followed at a leading pediatric medical center from 2001–2010 reveals no demonstrable changes in the epidemiology of this disorder. CPP remains a rare diagnosis, and though a neurogenic cause should be suspected and investigated, the majority of boys with CNS-CPP had known neurologic disease prior to CPP.

## Supporting information

S1 Fig Temporal relationship between the diagnoses of a brain neoplasm and CPP in 15 patients. Squares indicate diagnosis of a brain neoplasm and triangles indicate CPP diagnosis. White squares indicate CPP diagnosed after the brain neoplasm, while gray squares indicate CPP diagnosis prior to neoplasm diagnosis.
(TIF)

## Abbreviations

95CI: 95% confidence interval
BCH: Boston Children’s Hospital
BMI: body mass index
CNS-CPP: neurogenic central precocious puberty
CPP: central precocious puberty
CV: coefficient of variation
DHEAS: dehydroepiandrosterone sulfate
FSH: follicle stimulating hormone
HPLC: high performance liquid chromatography
ICD-9: International Statistical Classification of Diseases 9^th^ edition
iCPP: idiopathic central precocious puberty
LOD: limit of detection
LOQ: limit of quantification
LH: luteinizing hormone
NF-1: Neurofibromatosis type 1
NHW: non-Hispanic White
PP: precocious puberty
QCS: quality control sera
SD: standard deviation
TT: total testosterone
