# Supplementary figures and images for: Central precocious puberty in Boston boys: A 10-year single center experience
Source: PLoS One. 2018 Jun 27;13(6):e0199019. doi: 10.1371/journal.pone.0199019 (PMC6021063; doi:10.1371/journal.pone.0199019)

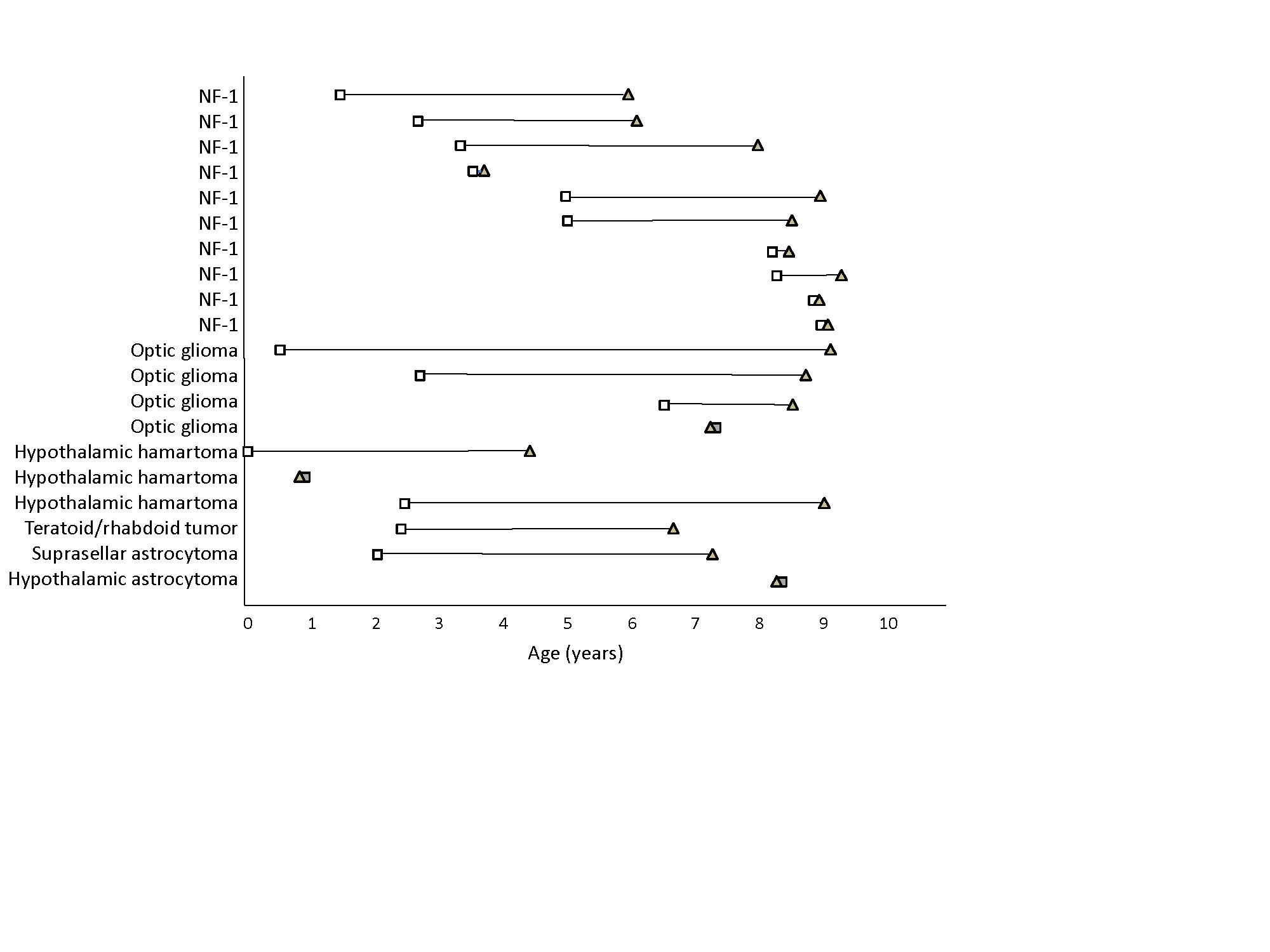

Supplement: S1 Fig — Squares indicate diagnosis of a brain neoplasm and triangles indicate CPP diagnosis. White squares indicate CPP diagnosed after the brain neoplasm, while gray squares indicate CPP diagnosis prior to neoplasm diagnosis. (TIF) [file pone.0199019.s001.tif]
